# Bayesian Edge Detector Using Deformable Directivity-Aware Sampling Window

**DOI:** 10.3390/e22101080

**Published:** 2020-09-25

**Authors:** Ren-Jie Huang, Jung-Hua Wang, Chun-Shun Tseng, Zhe-Wei Tu, Kai-Chun Chiang

**Affiliations:** 1 Department of Electrical Engineering, National Taiwan Ocean University, Keelung City 20224, Taiwan; 20553001@email.ntou.edu.tw (R.-J.H.); jjay6twtw@gmail.com (Z.-W.T.); 2 Deputy, AI Research Center, National Taiwan Ocean University, Keelung City 20224, Taiwan; 3 Ship and Ocean Industries R&D Center (SOIC), New Taipei City 25170, Taiwan; cstseng2954@gmail.com; 4 Department of Fine Arts, Taipei National University of the Arts, Taipei City 11201, Taiwan; kaichun.chiang@finearts.tnua.edu.tw

**Keywords:** Bayesian, edge detector, entropy, gestalt theory, EM algorithm

## Abstract

Conventional image entropy merely involves the overall pixel intensity statistics which cannot respond to intensity patterns over spatial domain. However, spatial distribution of pixel intensity is definitely crucial to any biological or computer vision system, and that is why gestalt grouping rules involve using features of both aspects. Recently, the increasing integration of knowledge from gestalt research into visualization-related techniques has fundamentally altered both fields, offering not only new research questions, but also new ways of solving existing issues. This paper presents a Bayesian edge detector called *GestEdge*, which is effective in detecting gestalt edges, especially useful for forming object boundaries as perceived by human eyes. *GestEdge* is characterized by employing a directivity-aware sampling window or mask that iteratively deforms to *probe* or *explore* the existence of principal direction of sampling pixels; when convergence is reached, the window covers pixels best representing the directivity in compliance with the similarity and proximity laws in gestalt theory. During the iterative process based on the unsupervised Expectation-Minimization (EM) algorithm, the shape of the sampling window is optimally adjusted. Such a deformable window allows us to exploit the similarity and proximity among the sampled pixels. Comparisons between *GestEdge* and other edge detectors are shown to justify the effectiveness of *GestEdge* in extracting the gestalt edges.

## 1. Introduction

**Figure 1:**
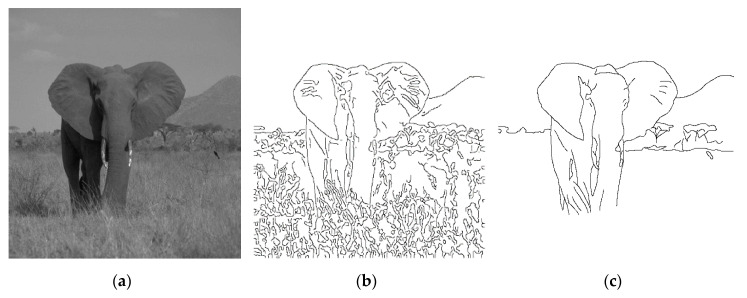
(**a**) A complex nature image. (**b**) Result of applying Canny detector to (**a**). (**c**) Object contours perceived by the human vision system.

The terms of edge and contour are often used interchangeably in the field of image processing. Still, the term “edge” is mostly used to denote image points where intensity difference between pixels is significant. On the other hand, the term “contour” is used to denote connected object boundaries. The goal of edge detection is to identify pixels at which the intensity or brightness changes sharply. Ideally, edge detection should generate a set of straight or curved line segments for defining some closed object boundaries, thus benefiting diverse research areas such as image segmentation [1], pattern recognition [2], and motion tracking [3,4]. Traditional edge detectors like Roberts [5] can hardly produce a set of connected lines and curves that correspond to object boundaries. Using low level edge detectors often yields redundant details and even false contours. These undesirable effects are particularly unacceptable wherein gestalt edges are required and grouped for constructing boundaries of objects as perceived by the human eyes [6], as the elephant, tress, and mountain in Figure 1c. Although Laplacian [7] and Canny [8] can be used to detect most edges by proper parameters adjustment, it is rather difficult (if not impossible) to produce gestalt edges, as they are based on differential calculation or criterion-based optimization. Figure 1b shows that too many fine edges or redundant details are extracted by the well-known Canny detector, particularly for the complex nature image of Figure 1a. The human visual system can quickly compile complex scenes into simple object contours for survival or even art creation; this observation should allow us to conjecture that successful detection of gestalt edges is not only useful for simplifying the low-level tasks of edge linking in forming closed contours, but also beneficial for the high-level operations of image analysis and even artwork creation.

The word “gestalt” is German for “unified whole”. Historically, the first gestalt principles were devised in the 1920s by psychologists Wertheimer, Koffka, and Kohler, who aimed to understand how humans typically gain meaningful perceptions from the chaotic stimuli around them. They identified a set of laws which address the natural compulsion to find order in disorder. The gestalt laws are a set of principles [9] to account for the observation that humans naturally perceive objects as organized patterns and objects. Gestalt psychologists argued that the human mind innately tends to perceive patterns in the stimulus based on certain rules. Normally, gestalt principles are organized into five categories: proximity, similarity, continuity, closure, and connectedness. The principle of similarity says that elements that are similar are perceived to be more related than elements that are dissimilar. Similarity helps us organize objects by their relatedness to other objects within a group and can be affected by the attributes of color, size, shape, and orientation. The law of proximity states that items that are close together tend to be perceived as a unified group. Namely, items close to each other tend to be grouped together, whereas items farther apart are less likely to be grouped together.

This paper associates gestalt theory, in particular the laws of proximity and similarity, together with the Expectation-Minimization (EM) algorithm and the Bayesian decision to achieve the extraction of gestalt edges for an input image. We present a novel method called *GestEdge*, in which the directivity of a target pixel is iteratively evaluated with a sampling window of which the shape is deformable by the EM algorithm. Upon convergence, the final directivity value should reflect the likelihood of pointing to the similar direction among the neighboring pixels within the converged window, and can be plugged into a Bayesian decision formula to determine whether the target pixel is qualified to be a gestalt edge point. In view of entropy as an inverse indicator of direction uniformity, plus the observation that the gradient always points in the direction of largest possible intensity increase and the length of the gradient vector corresponds to the rate of change in that direction, the deformable window enables *GestEdge* to exploit the proximity and similarity for each target pixel points. By sliding the detection window, left-to-right and top-to-bottom, through the entire input image, *GestEdge* can effectively detect gestalt edges essential for constructing contours consistent with human perception. The proposed method mainly comprises the following steps: (i) First, a subset of pixels is selected from the input image as POI (pixels of interest); (ii) then we take each pixel of the POI as a target pixel and iteratively update the shape of a detection window center at the target pixel; when convergence is reached, a directivity value representing the likelihood of perceiving the target pixel as edge point is obtained; (iii) then we invoke the Bayesian process [10], to determine whether the target pixel is a gestalt edge, and if it is not, the target pixel is eliminated; (iv) we then slide the window to the next pixel in POI and go to Step (ii), until all pixels in POI are processed; and, finally, (v) the remaining candidate pixels are outputted as the gestalt edge pixels.

## 2. Theoretical Basis

To help readers understand the theoretical basis of *GestEdge*, some assumptions pertaining to human mind disposition are elaborated as follows: (a) Assumption-1, which corresponds to the aforementioned similarity law, states that humans tend to notice neighboring pixels with similar gradient orientations by converting the gradient of neighboring pixels to illusory representations. This phenomenon can be found in Reference [11], wherein the famous Müller–Lyer illusion is illustrated. (b) Assumption-2 states that humans evaluate gradient with a large receptive field, e.g., human mind can easily perceive the elephant body contours by ignoring the much smaller grass objects on the ground (Figure 1c). Accordingly, it is believed that, in addition to using a small detection window as used in precedent edge detectors, a large receptive field must also be employed in the human visual system, to perform necessary operations for ignoring the grass on the ground. (c) Assumption-3 states that the human visual system perceives orientation similarities in a pixel-based fashion. Humans can easily perceive smooth varying contours, implying that a different mechanism from that of region-based detectors [12,13] must be employed.

Assumption-1 implies that the gradient orientation resemblance between neighboring pixels is essential to identify gestalt edges. As to Assumption-2, although numerous region-based methods [12,13] employ a large detection window to determine the principal orientations of pixels within the window, they often fail to capture smoothly varying contours. In contrast, this paper aims to embody the three assumptions simultaneously, so as to facilitate the extraction of gestalt edges. We propose a statistical approach called directivity-aware directivity scheme, wherein a deformable window is used to effectively evaluate the orientation resemblance between neighboring pixels. Assumption-1 and the proximity law require that if a target pixel is more similar (in terms of gradient orientation) and closer to its neighboring pixels, the more likely it is a gestalt edge pixel. Our experiment results show that *GestEdge* can detect, through the Bayesian decision on the directivity-aware directivity evaluation result, edge pixels that are ready for constructing object contours as perceived by humans.

## 3. Selection of Candidate Pixels

Initially, a low-pass Gaussian filter [14,15] is applied to the input, to obtain the output image *I* (*H* × *W* pixels, *x* = 1, 2, …, *H*, and *y* = 1, 2, …, *W*). Subsequently, gradients $g_{x}$ and $g_{y}$ are obtained by applying any gradient operator, such as a Sobel, Roberts, or Prwitt, to *I*. The gradient magnitude matrix, g, and the orientation matrix, ***θ****,* are calculated by using $\mathrm{formulas}\;\sqrt{g_{x}^{2}+g_{y}^{2}}$ and $arctan\left(g_{y}/g_{x}\right)$, respectively. Then, a normalized $g^{L}\left(x,y\right)$ is calculated by setting the largest and smallest element in log(g) to 1 and 0, respectively. In analogy to the non-maximum suppression (NMS) technique, for any pixel (x,y) with $g^{L}\left(x,y\right)<0.5$, its value of θ(x,y) can be simply replaced with an angle value randomly chosen from a uniform distribution over (−90, 90). Doing so is to neglect insignificant pixel intensity changes. Moreover, considering the fact that gestalt edges must be edges, yet the converse is not always true, for our purpose here, it is sufficient to use the detection result of any effective edge detectors as pixels of interest or candidate pixels. Without loss of generality, MATLAB-version Canny detector with double thresholds (0.25 and 0.1) [14] was used to produce pixels of interest (POI) in this work. As such, the subset of $g^{L}$ containing gradient magnitudes of pixels detected by the Canny detector is specifically denoted as $g_{Can}^{L}$.

## 4. Directivity-Aware Directivity Evaluation

**Figure 2:**
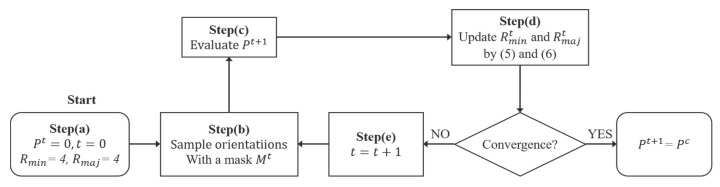
Flowchart of the entropy-driven directivity evaluation scheme.

The flowchart of *GestEdge* is shown in Figure 2, mainly comprising five steps.

**Step (a):** Quantize θ, using the formula $\left[\frac{\theta \times Bn}{180}\right]\times \left[\frac{180}{Bn}\right]$, where *Bn* is the user-specified total number of bins in the histogram of quantized ***θ***, and [l] denotes the integer nearest to l. Next, define a circular mask with an area of *r^2^* × π as the sampling window centered at a target pixel picked from POI and let the initial directivity $P^{t=0}\left(x,y\right)$ be zero. Heuristically, setting *r* to 4 (pixels) and, hence, *Bn* to 51 is sufficient for dealing with various types of images. The initial sampling window $M^{t=0}$ has a semimajor axis of 4 and a semiminor axis of 4. During the iteration, $M^{t}$ will rotate to align the semiminor axis with the quantized θ(x,y). Namely, the sampling window is set in parallel with the direction θ(x,y)+ 90°.

**Step (b):** This step, along with Step (c), corresponds to the E-step in the EM algorithm. Elements of θ covered by $M^{t}$ act as the observable data and are used for computing a histogram of gradient orientation, in which the height of a bin is written as $h_{i}^{t}\left(x,y\right)$. In particular, $h_{max}^{t}\left(x,y\right)$ denotes the *highest* bin among all bins $h_{i}^{t}\left(x,y\right)$, *i* = 1, 2… *Bn*. In a sense, $h_{max}^{t}\left(x,y\right)$ corresponds to the principal orientation in Reference [16]. Specifically, denote $h_{i}^{t}\left(x,y\right)$ as the bin associated with the target pixel.

**Step (c):** The directivity of the target pixel is updated as follows:

(1) $$P^{t+1}\left(x,y\right)=1-\frac{\left(S^{t}\left(x,y\right)+S_{bias}^{t}\left(x,y\right)\right)}{S_{max}^{Bn}}$$

(2) $$S^{t}\left(x,y\right)=-\overset{Bn}{\underset{i=1}{\sum }}h_{i}^{t}\left(x,y\right)log\left(h_{i}^{t}\left(x,y\right)\right)$$

(3) $$S_{bias}^{t}\left(x,y\right)=\left(1-\alpha \right)-S_{max}^{t}\left(x,y\right)-S^{t}\left(x,y\right)$$

In Equation (3), the parameter *α* is defined as $h_{T}^{t}\left(x,y\right)/h_{max}^{t}\left(x,y\right),$ and (1−α) measures the difference in the occurrence frequency between the gradient direction of the target pixel and the principal orientation. The term (1−α) interestingly has special implications for human visual perception, and we later elaborate on this further. Moreover, $S_{max}^{t}$ denotes the local maximum entropy:

(4) $$S_{max}^{t}=-\log\left(\frac{1}{q^{t}}\right),$$

and the global maximum entropy, $S_{max}^{Bn}$, is obtained when $q^{t}$ = *Bn*, which occurs when each pixel in $M^{t}$ by itself is a separate nonzero bin.

**Step (d):** This step corresponds to the M-step in the EM algorithm for updating the latent parameters (i.e., convergent semimajor and semiminor axes of $M^{t}$). The original circle, $M^{t}$, might deform to an ellipse, and the semiminor and semimajor axes are updated by using the following equation:

(5) $$R_{min}^{t}\left(x,y\right)=\{\begin{array}{cc} & \lfloor r\left(1-P^{t+1}\left(x,y\right)\right)\rfloor ,\;if\;P{\left(x,y\right)}^{t+1}>P{\left(x,y\right)}^{t}\;and\;\lfloor r\left(1-P^{t+1}\left(x,y\right)\right)\rfloor >1\\ & \lceil r\left(1-P^{t+1}\left(x,y\right)\right)\rceil ,\;if\;P{\left(x,y\right)}^{t+1}<P{\left(x,y\right)}^{t\;}and\;\lceil r\left(1-P^{t+1}\left(x,y\right)\right)\rceil >1\\ 1, & \mathrm{otherwise}\; \end{array}$$

(6) $$R_{maj}^{t}\left(x,y\right)=\frac{r^{2}}{R_{min}^{t}\left(x,y\right)}$$

**Step (e):**t=t+1; iterate Steps (b)–(d) until convergence. The converged directivity is noted as $P^{c}\left(x,y\right)$.

**Figure 3:**
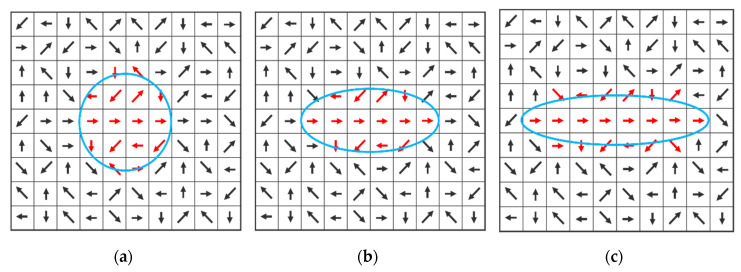
(**a**) $R_{min}$ = 4, $R_{maj}$ = 4; (**b**) $R_{min}$ = 2.6, $R_{maj}$ = 6; and (**c**) $R_{min}$ = 2, $R_{maj}$ = 8.

To facilitate understanding the flowchart of Figure 2, we use Figure 3 to schematically illustrate the deformation of sampling window $M^{t}$ as the directivity-aware scheme iterates Steps (b)–(d) until convergence. Figure 3a shows the initial $M^{t=0}$. After the first iteration of Steps (b)–(d), the sampling window is forced to deform by the zero-degree directivity evaluated at the center pixel in the first iteration. Figure 3c shows that the window shape is further elongated after the second iteration, to reflect the actual situation in which the target pixel is at, that is, more neighboring pixels are found to be in line with the target pixel (i.e., obeying laws of proximity and similarity), making it possess a high directivity when reaching convergence at the third iteration. Note that the entropy $S^{t}\left(x,y\right)$ for each window is calculated by using Equation (2), and, without the deformable window design, it would merely account for pixel intensity entropy. The parameter $q^{t}$ (<*Bn*) denotes the total number of nonzero bins. $S^{t}\left(x,y\right)$ inversely stands for the orientation resemblance between the pixels covered by $M^{t}$. In other words, a larger $S^{t}\left(x,y\right)$ indicates a weaker directivity because of the more uniform distribution of $h^{t}\left(x,y\right)$, and vice versa. Thus, using $h^{t}\left(x,y\right)$ to compute $S^{t}\left(x,y\right)$ enables the orientation resemblance within $M^{t}$ to be evaluated conveniently, which simulates the similarity law of gestalt theory [17] stating the tendency to group items (e.g., pixels and edges) into meaningful contours, if they are similar in terms of shape, color, or texture. Despite these good properties, human perception is quite a complex task from the perspective of information theory, which could render $S^{t}\left(x,y\right)$ inadequate for accurately measuring the directivity of a target pixel in some special cases. To see this, assume $S_{bias}^{t}\left(x,y\right)=0$, and then Equation (1) is readily reduced to the following:

(7) $$P{\left(x,y\right)}^{t}=1-\frac{S^{t}\left(x,y\right)}{S_{max}^{Bn}}$$

**Figure 4:**
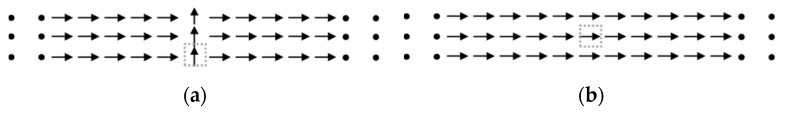
Two examples with an infinite number of samples: (**a**) a target pixel with a gradient orientation of 90° and (**b**) A target pixel with a gradient orientation of 0°.

Figure 4 shows two examples with an infinitely large receptive field. The target pixels are enclosed by dashed squares, with symbols → and ↑ representing orientations 0° and 90°, respectively. Figure 4a can be easily perceived as separate lines broken at the target pixel, whereas Figure 4b will be perceived as straight lines. Figure 4a,b are perceived differently, yet both cases have $S^{t}\left(x,y\right)=0$, using (2), because $h_{1}^{t}\left(x,y\right)=1$. That is, the normalized occurrence frequencies of → in Figure 4a,b are calculated as $\underset{n\to \infty }{\lim}\left(\frac{n-3}{n}\right)$ and $\underset{n\to \infty }{\lim}\left(\frac{n}{n}\right)$, respectively. Moreover, $h_{2}^{t}\left(x,y\right)=0$ (i.e., the normalized occurrence frequencies of ↑ in Figure 4a,b are calculated as $\underset{n\to \infty }{\lim}\left(\frac{3}{n}\right)$ and $\underset{n\to \infty }{\lim}\left(\frac{0}{n}\right)$, respectively. Using Equation (7), the directivity value for both target pixels in Figure 4a,b is 1, which is contradictory to human perception. To address this issue, we first note that the target pixel ↑ in Figure 4a should possess a directivity much smaller than that in Figure 4b. Clearly, a compensation term is required in Equation (7). In this study, the compensation term $S_{bias}^{t}$ is given as in Equation (3). In particular, (1−α) is defined as conflict index and has two implications: (i) $S_{bias}^{t}$ is regulated by α if $h_{max}^{t}\left(x,y\right)>h_{T}^{t}\left(x,y\right)$ or even if $h_{max}^{t}\left(x,y\right)$ equals approximately one. (ii) If $h_{max}^{t}\left(x,y\right)$ equals or is close to $h_{T}^{t}\left(x,y\right)$, then α≈0, and $S_{bias}^{t}$ is unnecessary.

For the first implication, we assume $h_{max}^{t}\left(x,y\right)\approx 1$ and $h_{T}^{t}\left(x,y\right)\approx 0$, which corresponds to Figure 4a, where many sampled pixels share the same orientation (i.e., 0°). In other words, a nearly zero entropy $S^{t}\left(x,y\right)$ indicates the existence of a dominant mode. For a large conflict index (≈1), a large value of $S_{bias}^{t}$ is required to get rid of the adverse effect. From Equation (3), $S_{bias}^{t}\approx S_{max}^{t}$, because $S^{t}\left(x,y\right)\approx 0$, and a small directivity value can be correctly obtained by using Equation (1). Thus, the problem in Figure 4a is solved. Note that, as the number of ↑ increases in Figure 4a, the value of (1−α) decreases, and as the number of ↑ goes to infinite, the conflict effect goes away, which is precisely the situation in Figure 4b. Clearly, $S_{bias}^{t}\left(x,y\right)$ can appropriately offset the conflict effect. That is, a larger value of $S_{bias}^{t}\left(x,y\right)$ can be obtained by using Equation (3) for offsetting a stronger conflict effect. The second implication simply states that either $h_{T}^{t}\left(x,y\right)$ itself is the dominant mode (e.g., 0° in Figure 4b) or at least two major modes coexist (i.e., $h_{max}^{t}\left(x,y\right)\approx h_{T}^{t}\left(x,y\right)$). In both cases,$\;S_{bias}^{t}$ is nearly zero, and the conflict effect is insignificant, making Equation (7) essentially identical to Equation (1).

**Figure 5:**
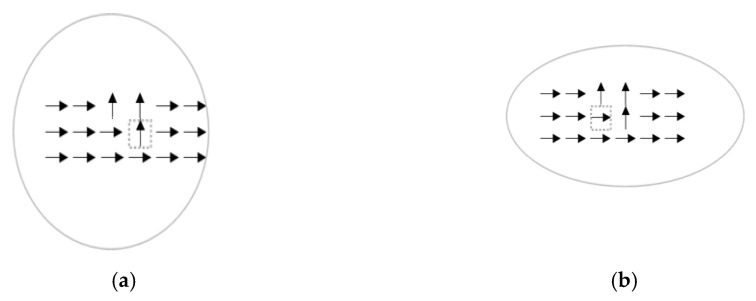
The window becomes narrower (wider) with a larger (smaller) directivity value. (**a**) A target pixel having a low directivity. (**b**) A target pixel having a high directivity.

Recall that $R_{min}^{t}\left(x,y\right)$ is rotated to align with θ(x,y) under the iterative EM algorithm. The window shape will become narrower (wider) for a larger (smaller) $P^{t}\left(x,y\right)$ value. Specifically, the window shape is allowed to deform iteratively, until it covers pixels from which a directivity value that best characterizes the target pixel can be evaluated. Doing so can simultaneously support the proximity and similarity laws, meaning that the EM-driven deformation not only enables as many pixels (with orientations similar to that of the target pixel) as possible to be covered by $M^{t}$, but also allows the target pixel to *spatially* depart from pixels that are dissimilar in gradient orientation. Figure 5a shows an example of a converged window centered at a target pixel of 90° (↑) when α = 0.2 (three pixels of ↑ and fifteen pixels of →; i.e., $h_{T}^{t}\left(x,y\right)$ = 3 and $h_{max}^{t}\left(x,y\right)$ = 15). In contrast, Figure 5b shows an example of a converged window centered at a target pixel of 0° (→) when α≈1 (i.e., $h_{T}^{t}\left(x,y\right)=h_{max}^{t}\left(x,y\right)=15$). From Equation (1), the directivity value of the target pixel (enclosed by a square) in Figure 5a is 0.05, which is smaller than the directivity value (0.36, using Equation (7)) of the target pixel in Figure 5b.

It is interesting to note that depictions in Figure 5a,b actually share the same histogram distribution, yet their converged window shapes are quite different. The high directivity possessed by the target pixel in Figure 5b indicates that if the target pixel is a gestalt pixel, the likelihood of observing such a histogram should be high; conversely, if a target pixel possesses a low directivity value, as in Figure 5a, then it should be very unlikely to observe such a histogram associated with a gestalt pixel. Therefore, a window deformed according to Equations (5) and (6) indeed is effective in measuring the likelihood of observing a gestalt pixel. In the context of directivity-awareness, although the target pixel in Figure 5a satisfies the proximity law that describes the gestalt tendency to group items into meaningful configurations [17], it does not meet the similarity law, and, hence, results in a low directivity value. In contrast, because the target pixel in Figure 5b satisfies both the proximity law and similarity law, it has a high directivity value and the converged window shape is much more elongated than that in Figure 5a. Comparison of Figure 5a,b justifies that our sampling window is directivity-aware in the sense that its deformation implicitly accounts for spatial occupation entropy and indeed can support both laws of proximity and similarity.

To prove the stability of the iterative directivity-aware scheme, an energy function is defined as follows:

(8) $$E^{t}\left(x,y\right)=-{\left[R_{min}^{t}\left(x,y\right)-r\left(1-P^{t+1}\left(x,y\right)\right)\right]}^{2}$$

The first derivative of *E^t^* with respect to *P^t^* can be written as

(9) $$∆E^{t}\left(x,y\right)=-2r\times \left[R_{min}^{t}\left(x,y\right)-r\left(1-P^{t+1}\left(x,y\right)\right)\right]∆P^{t}\left(x,y\right)$$

where $∆P^{t}\left(x,y\right)=P{\left(x,y\right)}^{t+1}-P{\left(x,y\right)}^{t}$. According to Equation (5), the term $\left[R_{min}^{t}\left(x,y\right)-r\left(1-P^{t+1}\left(x,y\right)\right)\right]$ in Equation (9) is always smaller than zero if $∆P^{t}\left(x,y\right)<0$, and it will be always greater than zero if $∆P^{t}\left(x,y\right)>0$. Namely, $∆E^{t}\left(x,y\right)$ is always negative. Therefore, $E^{t}\left(x,y\right)$ in Equation (8) is guaranteed to converge at least to a local minimum when $P^{t}\left(x,y\right)$ is iteratively updated. An analogy of this convergent process can be found in the well-known Hopfield Network [18] in updating the connection weights between neurons.

## 5. Determination of Gestalt Pixels

Given $P^{C}\left(x,y\right)$ and $g_{Can}^{L}\left(x,y\right)$, the decision regarding a gestalt pixel can be simply made using Bayesian formula with two separate classes: a class of gestalt pixels and the rest. In an extreme case, all pixels in $\mathrm{the}\;\mathrm{subset}\;\mathrm{of}\;g_{Can}^{L}$ belong to the class of gestalt pixels. For simplicity, $g_{Can}^{L}\left(x,y\right)$ can be conveniently taken as the prior probability of the class of gestalt pixels. The convergent directivity $P^{C}\left(x,y\right)$ is treated as the likelihood of the target pixel (x,y) being located on a gestalt edge. Namely, $P^{C}\left(x,y\right)$ is the probability of observing the event $h^{C}\left(x,y\right),$ given the fact that the target pixel (x,y) comes from the class of gestalt pixels. the posterior probability of a target pixel being on the gestalt edge can be approximately calculated as follows:

(10) $$Pcep\left(x,y\right)\approx \frac{P^{C}\left(x,y\right)\times g_{Can}^{L}{\left(x,y\right)}^{1/k}}{P^{C}\left(x,y\right)\times g_{Can}^{L}{\left(x,y\right)}^{1/k}+\left[\left(1-P^{C}\left(x,y\right)\right)\times \left(1-g_{Can}^{L}{\left(x,y\right)}^{1/k}\right)\right]}$$

where *k* serves as a control parameter, o < *k* ≤ 1. With *k* = 1, $g_{Can}^{L}{\left(x,y\right)}^{1/k}$ corresponds to the upper-bound of the prior probability of gestalt pixels covered by $M^{t}$. The Bayesian decision on whether the target pixel is gestalt can be simply made by using the following rule: If Pcep(x,y)>0.5, the pixel at (x,y) is determined to be a gestalt pixel. We note that, in Equation (10), the smaller the value of *k*, the smaller the value of Pcep(x,y), and, hence, the less likely the target pixel will be accepted as a gestalt pixel. After every pixel in Canny edges has been processed, the gestalt edges of *I* are obtained.

## 6. Experimental Results

### 6.1. Part 1: Nature Images

In References [19,20], the authors added a computational step of surround suppression to the Canny edge detector and a Gabor-based contour operator. Their resulting operators responded strongly to isolated lines and edges, region boundaries and object contours, and exhibited weaker or no responses to texture. Thus, it would be interesting to compare their results with *GestEdge*. Forty images of Reference [19] that consist of complex details (e.g., rivers, rocks, and bushes) and meaningful objects were used as input images for testing various edge detectors. FOM (figure of merit) [20] was adopted to measure the matching degree between the ground truth and the detection result. The matching ratio was obtained by averaging the corresponding scores over the 40 test images used in Reference [19]. The matching ratio of *GestEdge* with the Canny selection of pixels of interest is 0.42, in contrast to 0.39, 0.33, and 0.13 for References [19,20], with the Canny detector alone, respectively.

**Figure 6:**
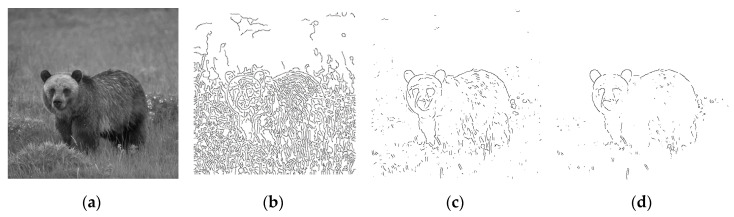
(**a**) Input image. (**b**) Detection result of applying Canny to (**a**). (**c**) Gaussian Filter [14] as preprocess prior to applying *GestEdge* (*k* = 1). (**d**) Adaptive Gaussian filter [15] as preprocess prior to applying *GestEdge* (*k* = 1).

**Figure 7:**
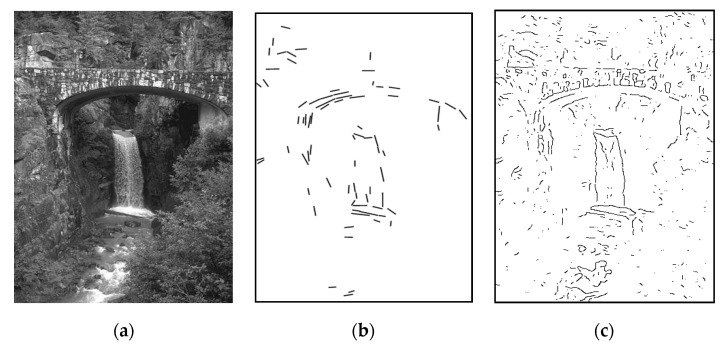
(**a**) A complex nature input image. (**b**) Contours depicted by applying LSD [13] to Figure 1a. (**c**) Contours depicted by applying our method to Figure 1a, *k* = 1.

**Figure 8:**
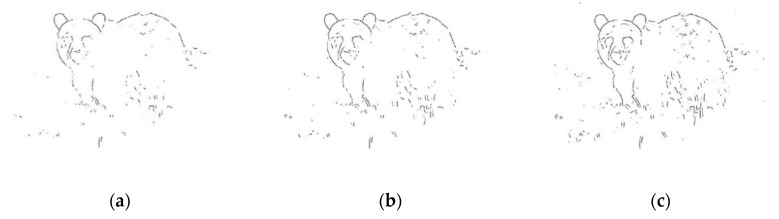
Comparison of using different *k* values and Gaussian Filter [14] as preprocess prior to applying *GestEdge*: (**a**) *k* = 0.5, (**b**) *k* = 0.6, and (**c**) *k* = 0.7.

More pictorial comparison results are presented in Figure 6. In comparison, Figure 6c preserves much more continuous contours than Figure 6b. Furthermore, using the adaptive Gaussian filter [15] as preprocessing prior to applying *GestEdge* shows the best result in Figure 6d. Using the complex nature image in Figure 7a as the test input, and comparing Figure 7b,c we can justify that *GestEdge* can preserve edges mostly close to the contours perceived by human vision in comparison to the LSD [13]. To see the effect of using different *k* values, Figure 8 shows the result of applying *GestEdge* to the input bear image in Figure 6a, with the Gaussian filter [14] as preprocess. As aforementioned, if the removal of less significant details, such as the grasses on the ground, is favored, while preserving only the contours of the bear, then a smaller value of *k* should be used; it is worth noting that the matching ratio of *GestEdge* can reach 0.48 if all input pixels are used as candidate pixels.

### 6.2. Part 2: Images Shot in Presence of Heavy Interferences

**Figure 9:**
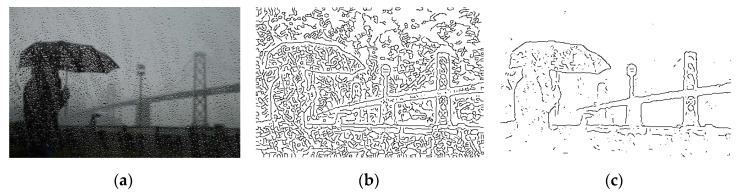
(**a**) Input image obscured by rain. (**b**) Detection result of applying Canny to (**a**). (**c**) Gaussian Filter [14] as preprocess prior to applying *GestEdge* (*k* = 1).

When tested with an input image obscured by rain, as shown in Figure 9a, *GestEdge* can still preserve major objects, i.e., the person, umbrella, and bridge, as if the rain does not cause any interferences.

### 6.3. Part 3: Rotoscoping

In the field of fine arts, the contour is the result of the artist’s personalized creation of hand-painted techniques and observation details. The invention of photography assisted the artist to grasp perspective and proportions more accurately. In the early days, it was thought that the creation of realistic style could be continued by using photography to imitate painting. At the end of the 19th century, a photographer named May Ray (1890–1976), a significant contributor to the Dada and Surrealist movements, used the photographic technique of solarization to create a clear shadow contour on the edges of black and white portraits. The work of May Ray reveals the possibility of using photography to illustrate contours; his work expands the logic of traditional sketching into the realm of photography. In the course of photography and motion pictures, the contour of the dynamic image has been paid attention to; one example is the technique of Rotoscope, which was first studied by Max Fleischer in 1915. This technique can be found in Walt Disney’s work *Snow White and the Seven Dwarfs* and its successive animated films. Based on the dynamic contours of real people, Walt Disney produced more natural 2D animated characters. Bob Sabiston (a director and programmer) created a programming language that simulates Rotoscope’s technology, simplifying the time and difficulty of production. In 2017, the film *Loving Vincent*, based on the story of Dutch artist Vincent van Gogh, also tried various methods for depicting the contours of characters. Despite these efforts, the technical difficulty of Rotoscope is that it must be done frame-by-frame, which is rather time-consuming and extremely costly.

**Figure 10:**
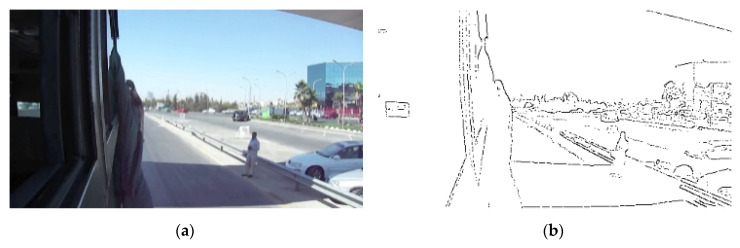

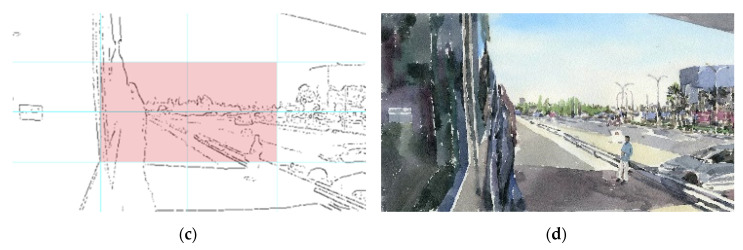
(**a**) Test image. (**b**) Result of *GestEdge*(*k* = 1). (**c**) The red block is the center of the picture, which is the emphasis of the picture. (**d**) Final colorized Rotoscoping work.

To exploit the feasibility of applying *GestEdge* in the field of visualization, the result of *GestEdge* tested on Figure 10a is shown in Figure 10b, and the colorized Rotoscoping work by an artist is shown in Figure 10d, for comparison. Some observations are given below:

(i) Figure 10b shows that the result of *GestEdge* can catch contours, shapes, and perspective in the landscape, pretty much encompassing all objective elements, such as buses, highway fences, distant forests, and close-up figures; these elements would also have been pinpointed by an artist using his/her own eyes.
(ii) High contrast areas of the original image are depicted by relatively non-fragmented edges in the *GestEdge* result. However, it failed to yield the same desirable performance for those low-contrast spots.
(iii) Distant details in Figure 10a are not well picked by *GestEdge*; they are only extracted as small points.
(iv) Aesthetic judgment: The red block corresponds to the central area of the image, which is likely to receive more attention, naturally, from the artist; that is, during the art creation, artists would normally increase bold lines and brush strokes, to highlight the difference between objects and the ground in this particular area.

Our method of quickly producing Rotoscoping can be directly applied to the work of Reference [21], where a 3D animation is edited by using the Rotoscoping technique, the result of which is then used as prior animation to produce a full training set of synthetic videos via perturbation of the original animation curves. Their test results on live videos lead to comparable accuracy, with the advantage of drastically reducing both the human effort and the computing power needed to produce the live training material.

**Figure 11:**
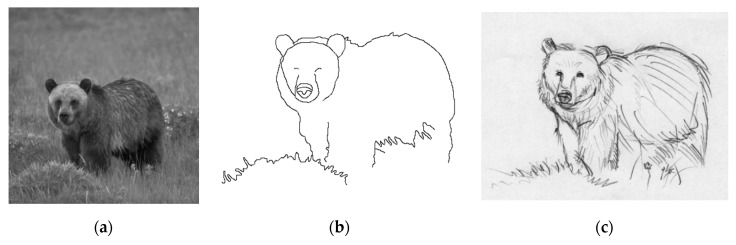
A rough sketch for drawing or animation: (**a**) original image, (**b**) ground truth in terms of gestalt theory, and (**c**) a rough sketch by artist.

The output of *GestEdge* can be used as a rough sketch (Figure 11c) for the subsequent Rotoscoping work of drawing or animation, thus helping the artist create the Rotoscoping work more easily, as with the one shown in Figure 10d. Finally, our work can be found useful in performing data augmentation for deep-learning applications. Data augmentation is often required in deep learning, especially when sufficient training data are either too costly or essentially unavailable. The rationale can be clearly seen from the work of Reference [22], wherein the Rotoscoping result is used as prior animation, to produce a full training set of synthetic videos, i.e., an implementation of data augmentation.

### 6.4. Part 4: Using Output of Superpixels as Candidate Pixels

**Figure 12:**
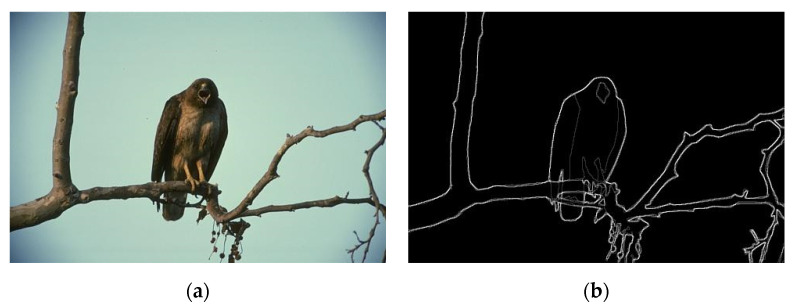
A test image selected from BSD300 [24]: (**a**) original image and (**b**) ground truth.

**Figure 13:**
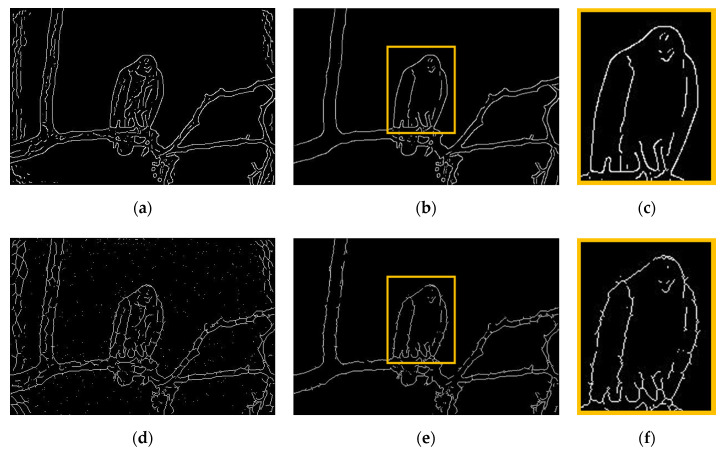
Comparison result between Canny and superpixels. (**a**) Canny detection as candidate pixels, applying *GestEdge* (*k* = 1) to Figure 12a. (**b**) The result of applying “AND” operator to dilated ground truth of Figure 12b and Figure 13a. (**c**) The orange block zoom in from (**b**), which is the emphasis of the picture. (**d**) Superpixels as candidate pixels, applying *GestEdge* (*k* = 1) to Figure 12a. (**e**) The result of applying “AND” operator to dilated ground truth of Figure 12b and Figure 13d. (**f**) The orange block zoom in from (**e**), which is the emphasis of the picture.

We also examined the performance of *GestEdge* by using a POI generator other than the Canny detector. We used the boundaries of superpixels generated by algorithms such as the context-aware superpixel (CASP) algorithm [23]. Just like *GestEdge*, CASP attempted to simulate similarity and proximity gestalt grouping rules by using bilateral entropy (BE) with conditional intensity entropy and spatial occupation entropy, aiming to generate superpixels in a fine-level. For simplicity, the MATLAB built-in function of *superpixels* in its image process toolbox was used (with the total number of superpixels = 500) to generate the candidate pixels. A natural image database BSD300 [24] containing pairs of the original image and the corresponding ground truth is suitable for our purpose here. Figure 12 is one test image pair, wherein the original image contains a background of sky and a foreground of one eagle standing on tree branches. Pictorial comparison results are shown in Figure 13. The output results of *GestEdge*, using Canny candidate pixels, are shown in Figure 13a, and that of *GestEdge*, using superpixels as candidate pixels, is shown in Figure 13d. Clearly, much more neat and tidy edges were preserved in Figure 13a than in Figure 13d. Furthermore, Figure 13b shows the result of applying “AND” operator to Figure 12b dilated by a diamond mask and Figure 13a, whereas Figure 13e shows the result of applying “AND” operator to Figure 12b dilated by a diamond mask and Figure 13d. To see performance difference more clearly, the zoom-in results of the orange rectangle in Figure 13c,f are shown in Figure 13b,e, respectively. We can see that, in Figure 13f, there are many tiny random fragmented edges that were preserved, and some of them were misjudged by *GestEdge* as perceptual edges, causing the contours to be a little bit more unsmooth than in Figure 13c. We believe this phenomenon may be caused by the fact that superpixels are inherently mesh-like, making it harder to fit the real contour of the target object.

## 7. Conclusions and Discussions

The advantages of *GestEdge* are fourfold: (i) With the iterative EM algorithm, the sampling window deforms to cover neighboring pixels optimally exhibiting the directivity of the target pixel. (ii) Unlike the Canny detector, which requires double thresholds to be properly set, our method determines whether a target pixel is a gestalt pixel simply based on the directivity value derived from the unsupervised EM algorithm and Bayes formula. (iii) With a small set of parameters, it is capable of simultaneously satisfying the gestalt laws of proximity and similarity. By contrast, none of the previous works [12,13,19,20] can achieve the same performance. (iv) Noise and outliers can be easily suppressed because they have smaller values of directivity.

In the future, we will also try using the resulting object boundary pixels generated by Joint Contour Filtering approach [25], as its filters can efficiently extract subjectively meaningful structure. Finally, in order to achieve satisfactory performance in applications such as computer vision, image segmentation, and visualization, it is desired to have a closed contour for the object in question; we will further improve *GestEdge* by incorporating the other three laws in gestalt theory, i.e., continuity, closure, and connectedness, into the design of the directivity-aware directivity scheme. For example, when an unprocessed target pixel is Bayesian decided as highly directive, then its neighboring pixels covered by the rather elongated window (i.e., $R_{min}=1or2)$ should undergo a checking process to see if they share the same or nearly identical gradient with the target pixel; if so, the qualified pixels will be connected with the target pixel and, at the same time, marked as “processed”.

## References

[1] Cai J., Miklavcic S.J. (2013). Surface fitting for individual image thresholding and beyond. IET Image Process..

[2] Shotton J., Blake A., Cipolla R. (2008). Multiscale Categorical Object Recognition Using Contour Fragments. IEEE Trans. Pattern Anal. Mach. Intell..

[3] Sanchez-Nielsen E., Hernández-Tejera M. (2011). Real-time tracking using A∗ heuristic search and template updating. IET Comput. Vis..

[4] Cai L., He L., Yamashita T., Xu Y., Zhao Y., Yang X. (2011). Robust Contour Tracking by Combining Region and Boundary Information. IEEE Trans. Circuits Syst. Video Technol..

[5] Roberts L.G. (1965). Machine Perception of Three-Dimensional Solids. Optical and Electro-Optical Information Processing.

[6] Treisman A.M., Gelade G. (1980). A feature-integration theory of attention. Cogn. Psychol..

[7] Gonzalez R.C., Woods R.E. (2007). Digital Image Processing.

[8] Canny J. (1986). A Computational Approach to Edge Detection. IEEE Trans. Pattern Anal. Mach. Intell..

[9] Todorovic D. (2008). Gestalt principles. Scholarpedia.

[10] Laplace P.S. (1814). Théorie Analytique des Probabilités.

[11] Müller-Lyer F.C. (1889). Optische urteilstäuschungen. Arch. für Anat. Physiol. Physiol. Abt..

[12] Desolneux A., Moisan L., Morel J. (2007). From Gestalt Theory to Image Analysis: A Probabilistic Approach.

[13] Von Gioi R., Jakubowicz J., Morel J.-M., Randall G. (2008). LSD: A Fast Line Segment Detector with a False Detection Control. IEEE Trans. Pattern Anal. Mach. Intell..

[14] McAndrew A., Wang J.H., Tseng C.S., Asia (2010). Introduction to Digital Image Processing with MATLAB.

[15] Gomez G. Local smoothness in terms of variance: The adaptive Gaussian filter. Proceedings of the British Machine Vision Conference 2000, BMVC 2000.

[16] Tseng C.S., Lin C.T., Lin C.W., Wang J.H. Gestalt Edges Preservation Conformal to Human Vision Perception. Proceedings of the IEEE 13th International Conference on Information Reuse and Integration (IRI 2012).

[17] King D.B., Wertheimer M. (2005). Max Wertheimer and Gestalt Theory.

[18] Hopfield J.J. (1982). Neural networks and physical systems with emergent collective computational abilities. Proc. Natl. Acad. Sci. USA.

[19] Grigorescu C., Petkov N., Westenberg M.A. (2004). Contour and boundary detection improved by surround suppression of texture edges. Image Vis. Comput..

[20] Papari G., Petkov N. (2011). An improved model for surround suppression by steerable filters and multilevel inhibition with application to contour detection. Pattern Recognit..

[21] Radford A., Metz L., Chintala S. (2015). Unsupervised representation learning with deep convolutional generative adversarial networks. arXiv.

[22] Covre N., Nunnari F., Fornaser A., Cecco M.D. Generation of action recognition training data through rotoscoping and augmentation of synthetic animations. Proceedings of the 6th International Conference on Augmented Reality, Virtual Reality and Computer Graphics, AVR 2019.

[23] Liu F., Zhang X., Wang H., Feng J. (2020). Context-Aware Superpixel and Bilateral Entropy—Image Coherence Induces Less Entropy. Entropy.

[24] Martin D., Fowlkes C., Tal D., Malik J. A Database of Human Segmented Natural Images and its Application to Evaluating Segmentation Algorithms and Measuring Ecological Statistics. Proceedings of the IEEE International Conference on Computer Vision (ICCV).

[25] Wei X., Yang Q., Gong Y. (2018). Joint Contour Filtering. Int. J. Comput. Vis..

